# A Miniaturized Screen of a *Schistosoma mansoni* Serotonergic G Protein-Coupled Receptor Identifies Novel Classes of Parasite-Selective Inhibitors

**DOI:** 10.1371/journal.ppat.1005651

**Published:** 2016-05-17

**Authors:** John D. Chan, John D. McCorvy, Sreemoyee Acharya, Malcolm E. Johns, Timothy A. Day, Bryan L. Roth, Jonathan S. Marchant

**Affiliations:** 1 Department of Pharmacology, University of Minnesota, Minneapolis, Minnesota, United States of America; 2 Department of Pharmacology, University of North Carolina at Chapel Hill, Chapel Hill, North Carolina, United States of America; 3 Department of Biomedical Sciences, Iowa State University, Ames, Iowa, United States of America; 4 Division of Chemical Biology and Medicinal Chemistry, Eshelmann School of Pharmacy, University of North Carolina at Chapel Hill, Chapel Hill, North Carolina, United States of America; 5 National Institute of Mental Health Psychoactive Drug Screening Program (NIMH PDSP), School of Medicine, University of North Carolina at Chapel Hill, Chapel Hill, North Carolina, United States of America; 6 Stem Cell Institute, University of Minnesota, Minneapolis, Minnesota, United States of America; McGill University, CANADA

## Abstract

Schistosomiasis is a tropical parasitic disease afflicting ~200 million people worldwide and current therapy depends on a single drug (praziquantel) which exhibits several non-optimal features. These shortcomings underpin the need for next generation anthelmintics, but the process of validating physiologically relevant targets (‘target selection’) and pharmacologically profiling them is challenging. Remarkably, even though over a quarter of current human therapeutics target rhodopsin-like G protein coupled receptors (GPCRs), no library screen of a flatworm GPCR has yet been reported. Here, we have pharmacologically profiled a schistosome serotonergic GPCR (Sm.5HTR) implicated as a downstream modulator of PZQ efficacy, in a miniaturized screening assay compatible with high content screening. This approach employs a split luciferase based biosensor sensitive to cellular cAMP levels that resolves the proximal kinetics of GPCR modulation in intact cells. Data evidence a divergent pharmacological signature between the parasitic serotonergic receptor and the closest human GPCR homolog (Hs.5HTR7), supporting the feasibility of optimizing parasitic selective pharmacophores. New ligands, and chemical series, with potency and selectivity for Sm.5HTR over Hs.5HTR7 are identified *in vitro* and validated for *in vivo* efficacy against schistosomules and adult worms. Sm.5HTR also displayed a property resembling irreversible inactivation, a phenomenon discovered at Hs.5HTR7, which enhances the appeal of this abundantly expressed parasite GPCR as a target for anthelmintic ligand design. Overall, these data underscore the feasibility of profiling flatworm GPCRs in a high throughput screening format competent to resolve different classes of GPCR modulators. Further, these data underscore the promise of Sm.5HTR as a chemotherapeutically vulnerable node for development of next generation anthelmintics.

## Author Summary

Parasitic flatworms express a diverse array of G protein coupled receptors, but our knowledge of their pharmacological profile is limited. No high throughput screen of a flatworm GPCR has been reported, even though these targets have precedent for high druggability and functionality in the chemotherapeutically vulnerable excitable cell niche. The goal of this study was to establish a method for profiling flatworm G protein coupled receptors that can be scaled to high content screening. Using a cAMP biosensor, we have performed a proof of principle miniaturized screen on a schistosome serotonergic GPCR that resolves new ligands that potently and selectivity block 5-HT receptor activity *in vitro*, and 5-HT evoked responses in schistosomules and adult worms. This approach evidences the pharmacological divergence of a parasitic GPCR from the closest human homolog and a capacity for high content interrogation of flatworm GPCR properties and ligand specificities.

## Introduction

The neglected tropical disease Schistosomiasis is the most socioeconomically devastating helminth infection, and the second most burdensome parasitic infection behind malaria, infecting over 200 million people worldwide [1]. Infected individuals are treated by the drug praziquantel (PZQ), the mainstay therapeutic for disease control.

PZQ was originally developed during the 1970s, and the continued effectiveness of this agent over four decades of usage for treating a variety of parasitic infections has proven critically impactful [1]. Indeed this clinical efficacy has ironically proven to be a factor that has restrained efforts to develop alternative therapies, and at the most basic level, define how PZQ works. However several features of PZQ remain less than ideal and require improvement. First, our lack of mechanistic understanding of how PZQ works has proved a roadblock in the rational design of new drugs. There is a need to identify new druggable targets that exploit broader vulnerabilities within PZQ-sensitive pathways [2–4]. Second, our inability to improve on PZQ by chemical derivatization of the drug: all PZQ derivatives synthesized to date are less effective than the parent compound. The need is to identify novel structural pharmacophores that impair parasite viability. Third, the inability of PZQ to kill all parasitic life cycle stages. Juvenile worms are refractory to PZQ [5,6], possibly a contributory factor driving development of drug resistance [5,7]. The need is to identify new targets expressed throughout all lifecycle stages that are ideally conserved in other PZQ-sensitive parasites. Fourth, sub-optimal cure rates in the field: PZQ requires multiple drug dosings to achieve maximal cure rates for schistosomiasis, a regimen which is not always executed in mass drug administration efforts [8,9]. Therefore, there is clear opportunity to improve on the clinical penetrance of PZQ. These issues support efforts to identify new, druggable targets for development of next generation anthelmintics.

A logical place to start is with downstream effectors within the broader PZQ interactome. Over the last few years, therefore, our laboratory has attempted to bring fresh perspective to understand how PZQ works based upon a serendipitous basic science finding. During regeneration of the planarian flatworm *D*. *japonica*–a widely used regenerative biology model [10]–PZQ miscued polarity signaling to cause regeneration of bipolar (‘two-headed’) worms with dual, integrated organ systems [11]. This visually striking phenotype, coupled with the tractability of the planarian system to *in vivo* RNAi, allowed us to progressively define pathways engaged by PZQ *in vivo* [11–14]. These studies culminated in a model where PZQ acts as an ergomimetic [13] with *in vivo* PZQ efficacy regulated by the opposing functionality of dopaminergic and serotonergic neurons [11–14], known regulators of muscular activity, the tissue where planarian polarity determinants reside [15]. The serotonergic and dopaminergic G protein coupled receptors (GPCRs) engaged by activity of these bioaminergic neurons therefore represent potential downstream PZQ effectors. Their engagement by ligands, as shown for bromocriptine and other ergot alkaloids, phenocopy PZQ action *in vivo* [13,14].

This is an important realization as flatworm G protein coupled receptors (GPCRs) are logical candidates for antischistosomal drug development efforts. Over one quarter of current therapeutics target rhodopsin-like GPCRs [16]. However, barriers have been a lack of understanding of the physiology of specific GPCRs from within the broad GPCR portfolio (~75–120 in *S*. *mansoni* [17–19]) expressed by these organisms, as well as struggles optimizing functional expression of individual flatworm GPCRs in heterologous assay systems. However several groups have now begun to define a role for specific GPCRs within the chemotherapeutically vulnerable excitable cell niche [13,20–22], highlighting the key challenge of optimizing robust platforms for pharmacologically profiling these GPCRs in a miniaturized format compatible with high throughput screening (HTS). To our knowledge, no library screen of a flatworm GPCR has yet been reported. Prior studies have simply relied on interrogation of expressed GPCRs against handfuls of ligands selected around inferred agonist specificity.

Therefore the goal of this study was to establish a method for profiling flatworm GPCRs that can be effectively scaled to HTS. Our priorities for a platform were: first, a robustness for miniaturization into a multiwall plate format to permit chemical library screening, and second, use of a proximal readout of receptor activity within intact cells to enable real time monitoring of GPCR activity that can resolve different types of modulators (full, partial and inverse agonists, allosteric modulators). One technology that fulfills these requirements employs a bioluminescent cAMP reporter to monitor the activity of G_s_ and G_i_-coupled GPCRs, marketed as GloSensor. The assay is based upon a crucially permutated form of firefly luciferase incorporating a cAMP-binding domain from PKA, such that cAMP-binding causes a conformational change in the enzyme that enhances the luminescent signal [23]. The dynamic range and sensitivity of the biosensor has been shown to be compatible with a variety of HTS assays [23,24].

To evaluate this technology, we applied this approach to pharmacologically profile a *S*. *mansoni* serotonergic GPCR (Sm.5HTR) that has been shown *in vitro* to respond to 5-HT through elevation of cAMP [20]. Sm.5HTR is the parasitic homologue of the planarian serotonergic GPCR (S7.1) that we have recently shown modulates the efficacy of PZQ *in vivo* [13]. However, as with most flatworm GPCRs, little is known about the pharmacology of this receptor. An initial characterization revealed blockade of 5-HT evoked signals in the presence of high concentrations (100μM) of mammalian bioaminergic blockers [20]. Here, we have applied the GloSensor assay in a proof of principle pilot screen for flatworm GPCR modulation. Our data evidence the extent of pharmacological divergence between the schistosome receptor and the human 5-HT_7_-receptor homolog (Hs.5HT7R), and reveal new ligands and compound series selective for the parasitic GPCR. Finally, despite these differences in ligand selectivity, we demonstrate conservation of an unusual antagonist-evoked inactivation mechanism for Sm.5HTR, a pharmacological phenomenon also exhibited at Hs.5HT7R [25,26], where exposure to a subset of antagonists results in a prolonged inactivation of signaling activity from the receptor. This property enhances the attractiveness of Sm.5HTR as an anthelmintic drug target.

## Results

### Functional expression of a schistosome 5-HT receptor

In schistosome parasites, 5-HT is myoexcitatory: exogenous addition of 5-HT to schistosomules causes an increase in basal contractility and 5-HT also increases mobility of adult worms [27,28]. While this action has long been known, it is only in the last several years that the relevant receptors mediating the effects of 5-HT in flatworms have been identified [13,20]. The most abundant schistosome 5-HT receptor in adult worms from transcriptomic analysis [29], is a recently characterized GPCR christened Sm.5HTR [20].

**Fig 1:**
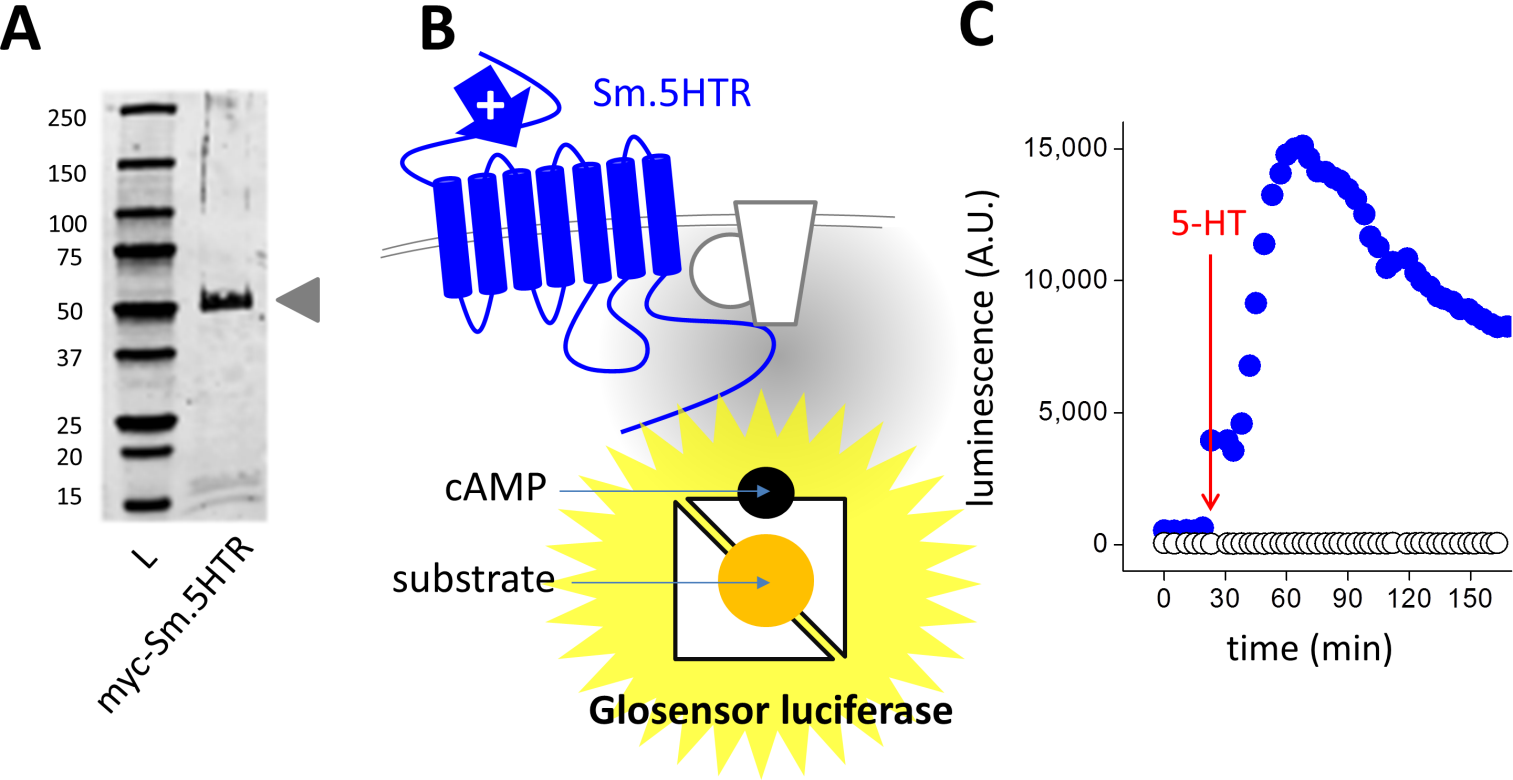
Functional expression of Sm.5HTR. (**A**) Western blot of myc-tagged Sm.5HTR (Genbank KF444051.1) in HEK293 cells. L, ladder. (**B**) Schematic of GloSensor assay depicting generation of cAMP by activation of Sm.5HTR (blue) which couples to endogenous G proteins and adenylate cyclase to increase cAMP levels. cAMP binding to the permuted luciferase construct leads to enhanced luminescence in the presence of substrate. (**C**) Functional expression of Sm.5HTR using this assay. In cells transfected with the biosensor (22F variant) addition of 5-HT (10μM) caused an increase in luminescence in Sm.5HTR transfected (blue symbols), but not control cells (open symbols).

Expression of an epitope tagged Sm.5HTR construct in HEK293 cells resulted in expression of a ~56 kDa product, consistent with the predicted size (Fig 1A). To assess functionality of this receptor, we utilized the GloSensor cAMP assay as a real-time luminescent readout of cellular cAMP levels. This ‘biosensor’ monitors luminescence from a firefly luciferase that is engineered to be cAMP sensitive by incorporation of a cAMP binding domain into the recombinant luciferase. The presence of substrate and cAMP results in an enhanced luminescence from the transfected GloSensor construct (Fig 1B), allowing real time monitoring of cAMP levels within intact cells. This can be seen in HEK293 cells transfected with both Sm.5HTR and GloSensor, where application of 5-HT evoked an increase in luminescence values over time (Fig 1C). No changes in cAMP were elicited in HEK293 cells transfected with the biosensor alone (Fig 1C).

### Optimization of assay conditions

**Fig 2:**
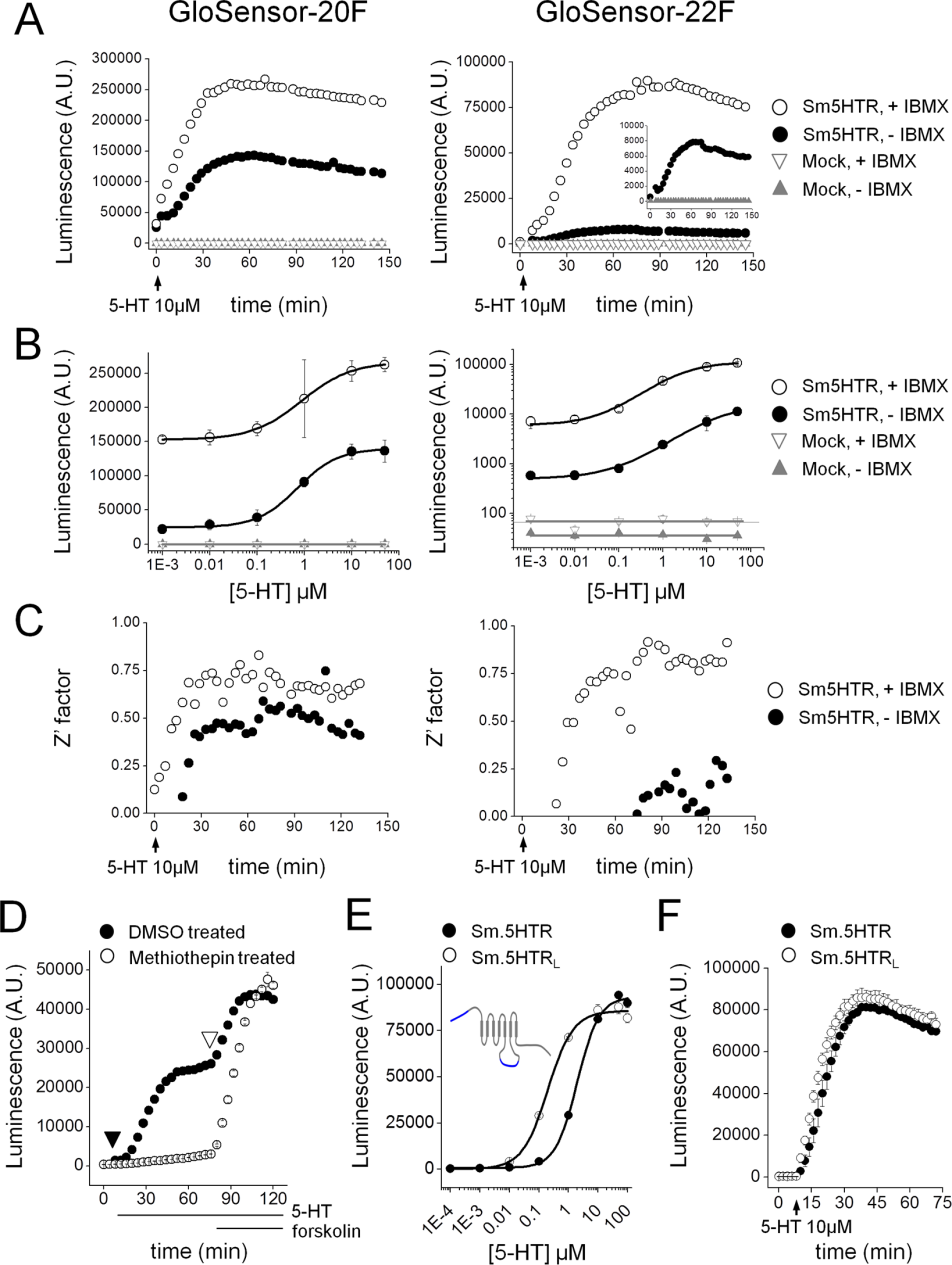
Optimization of cAMP bioluminescent sensor for monitoring Sm.5HTR activity. (**A**) Timecourse of cAMP changes following application of 5-HT (10μM) in HEK293 cells transfected with Sm.5HTR (circles) or untransfected controls (triangles). Assays are shown in the presence (open symbols) and absence (solid symbols) of a phosphodiesterase inhibitor (IBMX, 200μM). Luminescence values were recorded every 3 minutes in cells transfected with the high affinity (F20, left) or low affinity (F22, right) cAMP biosensor. Inset shows data for Sm.5HTR in the absence of IBMX on a rescaled y-axis. (**B**) 5-HT dose response curves from experiments such as those described in (A) for F20 (left) and F22 (right) in the presence (open) or absence (solid) of IBMX. Control data using untransfected cells are also shown (triangles). Note log scale for responses with 22F. (**C**) Z’ values calculated for 5-HT evoked signals over time under the conditions described previously. (**D**) Real time comparison of methiothepin pretreatment on 5-HT evoked changes in cAMP. One cohort of cells was preincubated with methiothepin (10μM, open circles), prior to addition of 5-HT (10μM) and IBMX (200μM, closed triangle) and then forskolin (20μM, open triangle). (**E**) 5-HT dose response curves for Sm.5HTR (solid) and Sm.5HTR_L_ (open). Inset, schematic comparison of schistosome 5HT receptor isoforms. Sm.5HTR_L_ is a longer isoform with additional sequence at the N-terminus and third intracellular loop. (**F**) Kinetics of 5-HT response (10μM) for Sm.5HTR and Sm.5HTR_L_.

Measurements of assay sensitivity were made from 5-HT evoked luminescence signals in cells plated in 96-well plates transfected with cAMP biosensors exhibiting either high affinity (‘F20’ construct) or low affinity (‘F22’ construct) for cAMP (Fig 2A). As expected, the magnitude of the luminescence signal varied with 5-HT application in a dose-dependent manner with both biosensor constructs in Sm.5HTR transfected cells (Fig 2B). With the higher affinity 20F sensor, the EC_50_ for cAMP generation was 703±90nM (n = 3, Fig 2B and Table 1), with the dose response relationship shifting to higher values with the 22F sensor as previously established [23]. The magnitude of the response was greatest in media supplemented with 3-isobutyl-1-methylxanthine (IBMX, 200μM) to block cAMP degradation. In the presence of IBMX, higher overall luminescence values were recorded with peak signal to background changes of ~1.7-fold and ~15.1-fold for 20F and 22F respectively (Fig 2A and 2B), providing a good signal to background window for monitoring receptor activation.

The robustness of these cAMP assays was assessed by calculating the Z’ factor (Z’), a widely used indicator of assay quality in high throughput screening applications [30]. Z’ values over 0.5 are considered a prerequisite for executing high throughput screens. Calculations of Z’ were made at different timepoints during the agonist response, averaging 6 replicate wells within a 96 well plate. In our hands, the highest Z’ scores were obtained with the F22 sensor supplemented with IBMX (Fig 2C), and these conditions were used for all subsequent assays. Acceptable Z’ values were also obtained with cells in suspension (Table 1) and under conditions of further miniaturization to 384-well plates (Fig A in S1 Text).

**Table 1:** Summary statistics for live cell GloSensor cAMP assays on Sm.5HTR. Z’ factor, signal window and EC_50_ were calculated at 60 minutes after delivery of 5-HT (10μM).

*Adherent Cells*	F20		F22	
	(-) IBMX	(+) IBMX	(-) IBMX	(+) IBMX
Z’ factor*	0.4	0.7	-0.1	0.7
Signal window**	3.1	3.6	-0.3	12.3
EC_50_	0.7 μM	0.9 μM	16.1 μM	2.0 μM
*Suspension Cells*	F20		F22	
	(-) IBMX	(+) IBMX	(-) IBMX	(+) IBMX
Z’ factor*	0.9	0.2	0.7	0.9
Signal window**	37.5	0.7	7.4	40.8
EC_50_	1.4 μM	1.0 μM	4.2 μM	3.8 μM

* Z’ factor calculated for vehicle control and 5-HT (10μM); Z’ = 1 - (3(stdev_max_ + stdev_min_) / (mean_max_—mean_min_).

** Signal window calculated for vehicle control and 5-HT (10μM), defined as SW = (mean_max_—mean_min_—3(stdev_max_ + stdev_min_)) / stdev_max_.

Unlike assays requiring cell lysis for fixed timepoint measurement, the live cell biosensor allowed real time monitoring of cellular cAMP levels throughout ongoing experimental manipulations. Fig 2D demonstrates antagonism of 5-HT stimulated cAMP generation by the antipsychotic methiothepin [31], with cellular responsiveness demonstrable by the subsequent addition of forskolin. Dose response analyses also confirmed preferential activation of Sm.5HTR by 5-HT compared with other bioaminergic agonists (Fig B in S1 Text). Finally, we used this assay to compare responsiveness from two different isoforms of Sm.5HTR which have been isolated–the originally published sequence Sm.5HTR [20] and a longer isoform (Sm.5HTR_L_) containing addition sequence at the NH_2_-terminus and within the third intracellular loop (Fig 2E, inset). Both isoforms were activated by 5-HT, with Sm.5HTR_L_ displaying ~10-fold greater sensitivity (EC_50_ 0.2±0.03μM vs 2.0±0.2μM, Fig 2E) but a similar kinetic response (Fig 2F).

### Pharmacological profiling of Sm.5HTR against a GPCR modulator library

Sequence homology identifies Sm.5HTR as a member of the SER7 clade of serotonin receptors, clustering with planarian S7 receptors [13] and with Hs.5HT7R as the closest human homolog [20]. To characterize the extent of pharmacological conservation between the parasite and human serotonin receptor, we used the miniaturized cAMP assay to screen a commercial GPCR compound library (~250 compounds) for inhibitors of these receptors. An inhibitor screen was prioritized simply because of the improved likelihood of detecting antagonists over agonists (need to exclude false positives from stimulation of endogenous receptors), and the utility of these agents for blocking parasite motility.

**Fig 3:**
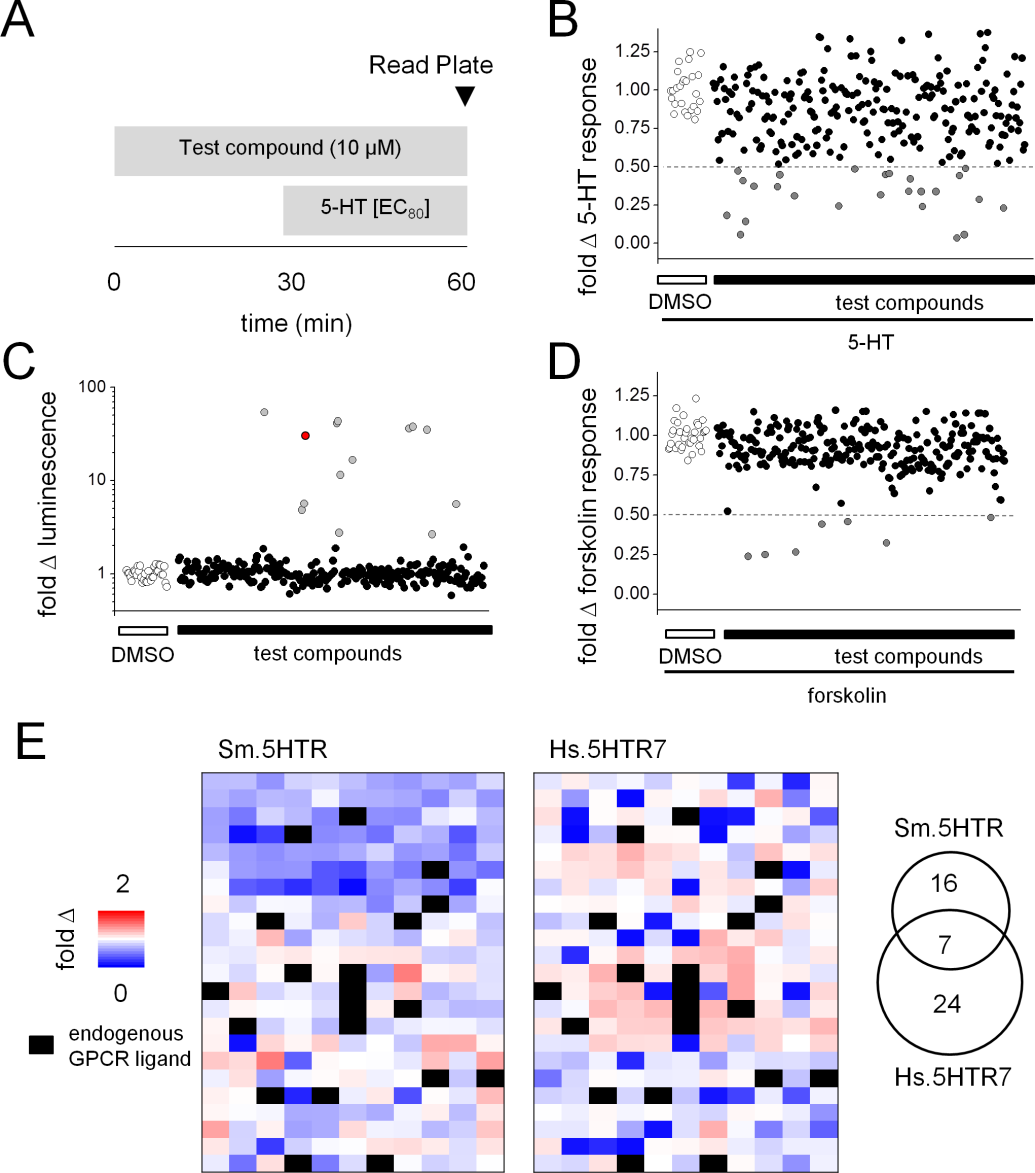
Pharmacological profiling of Sm.5HTR. (**A**) Schematic of assay workflow for screening a library of known GPCR ligands against HEK293 cells expressing either Sm.5HTR or Hs.5HT7. Cells transfected with either 5HTR and F22 cAMP biosensor were plated in 96-well format and exposed to test compounds (10μM). 5-HT was added after 30mins (at an EC_80_ concentration) after which luminescence values were recorded (time = 60min). (**B**) Scatter plots summarizing effects of test compounds on the Sm.5HTR response to 5-HT (dotted line highlights threshold for defining compound ‘hits’). Hits were defined at a threshold of ≥50% inhibition relative to control wells (DMSO only, open symbols). (**C**) Compounds were also screened against HEK293 cells expressing the F22 sensor alone (no Sm.5HTR) to screen for cAMP generation at endogenous receptors. For reference, a forskolin data point is shown in red. (**D**) Compounds were also screened against forskolin (20μM) evoked changes in luminescence relative to control samples (DMSO, open circles). (**E**) Heat map of all test compounds screened against *S*. *mansoni* Sm.5HTR (left) and human Hs.5HT7 (middle). Each colored box represents the fold change in luminescence in response to an individual test compound (253 in total) keyed by the pseudocolor scale. Compounds showing activity against endogenous receptors in cells transfected with the 22F biosensor alone (21 compounds total) were masked (black). Right, Venn diagram summarizing selectivity of antagonist ‘hits’ against either Sm.5HTR, Hs.5HT7R, or both 5-HT receptors. In total, 23 ligands were classified as potential ‘hits’ at Sm.5HTR and 31 ligands as ‘hits’ at Hs.5HTR7, with 7 in common.

The protocol for screening is shown schematically in Fig 3A. HEK293 cells transiently transfected with either the human 5HT_7_ receptor (Hs.5HT7R) or the schistosome receptor (Sm.5HTR) were exposed to test ligands in a 96-well plate format. After addition of test compounds, 5-HT was then added to each well at a concentration corresponding approximately to the EC_80_ of each receptor to assess blockade of 5-HT effects by prior compound addition. Luminescence was then read at a fixed time point (t = 60min, Fig 3A). Hits were assigned as compounds that evoked a ≥50% decrease in luminescence output at the fixed time sampling point (Fig 3B). These experiments identified 25 compounds as potential antagonists of Sm.5HTR evoked cAMP generation (Fig 3B).

Two sets of validation experiments were then performed in order to remove false ‘hits’ from the dataset. First, the same library was also screened against naive HEK293 cells as a control for responses resulting from engagement of endogenous GPCRs (Fig 3C). This analysis identified 14 compounds in the library that activated endogenous G_s_-coupled GPCRs in HEK293 cells [32]. Second, to exclude ligands that inhibited either cAMP production (for example, through activation of endogenous G_i_-coupled GPCRs) or directly impaired the activity of the luciferase biosensor, the library was screened against forskolin-evoked increases in cAMP (Fig 3D). This analysis identified 7 compounds that decreased luminescence values >2-fold in forskolin-treated control cells. These 21 compounds were ‘masked’ from the experimental dataset and the overall pharmacological profile of Sm.5HTR and Hs.5HTR7 were then represented as a heat map to depict ligand-evoked changes in cAMP levels (Fig 3E). This visual representation conveys in a simple manner the extent of pharmacological divergence between the human and schistosome GPCRs. Some drugs displayed a unique affinity for Sm.5HTRs, others preferentially modulated Hs.5HT7R, and some ligands blocked both receptors. Overall, 23 compounds were retained for subsequent validation as antagonists of Sm.5HTR with only a minor proportion of these compounds (7 ‘hits’) showing inhibition at both the human and parasite receptor (Fig 3E, inset). A simple overview of the pharmacological specificity of the compounds identified as antagonists using the ligand classification key associated with the library was also informative (Fig C in S1 Text). The types of ligand classes–if not compound identities–that inhibited each serotonergic GPCR was broadly similar. The only notable difference was blockade of Sm.5HTR by some cholinergic ligands, which was not apparent for Hs.5HT7R.

**Fig 4:**
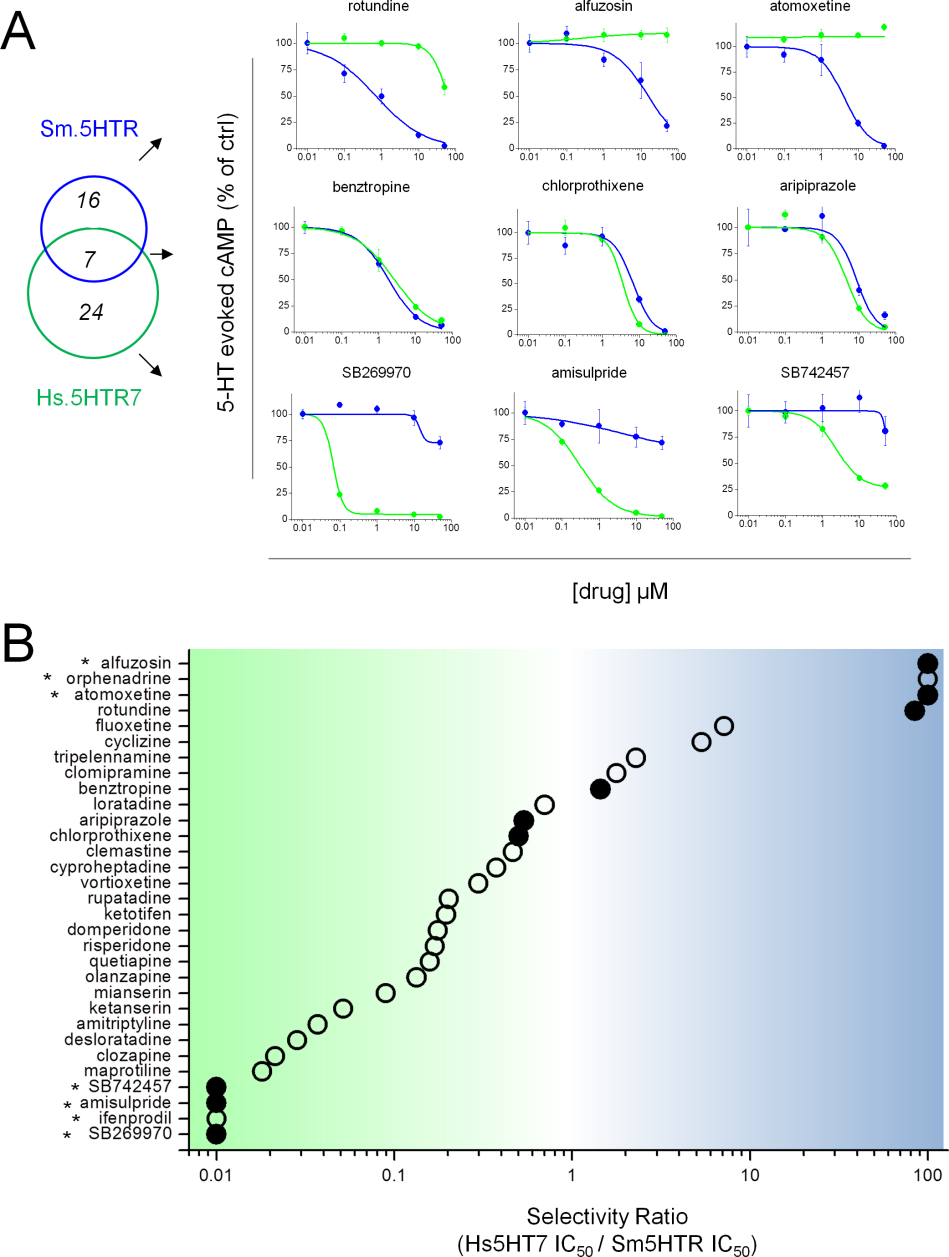
Profiling ligand selectivity for schistosome and human 5HT7 receptors. (**A**) Dose response relationships for compound antagonism of 5-HT evoked cAMP generation at human Hs.5HT7R (green) or parasite Sm.5HTR (blue) GPCRs. Illustrative data from compounds showing preferential selectivity toward Sm.5HTR (top) or Hs.5HT7R (bottom), or compounds with no selectivity (middle). Data represent mean±s.d, n = 3. (**B**) Schematic representation of ratio of IC_50_s for ‘hits’ profiled against both Sm.5HTR and Hs.5HT7R expressing HEK293 cells. Compounds exhibiting poor blockade of either GPCR (*) precluded calculation of IC_50_ values, so a minimal ratio estimate is provided. Solid circles represent compounds for which data is shown in ‘A’.

To confirm ‘hit’ validity, complete dose response relationships were then examined for all compounds that inhibited 5-HT evoked signals by ≥50%. Examples of these assays (Fig 4) confirm the designation of compounds showing selective inhibition of the parasite serotonin receptor (Fig 4A, top), blockade of 5-HT receptors from both species (Fig 4A, middle) and preferential antagonism of the human 5-HT receptor (Fig 4A, bottom). Calculation of a selectivity ratio (IC_50_ (Hs.5HT7R) / IC_50_ (Sm.5HTR)) for these antagonists (Fig 4B) revealed a broad continuum of GPCR selectivity among from the screened compounds. Four ligands demonstrated clear selectivity for Sm.5HTR (alfuzosin, orphenadrine, atomoxetine and rotundine, Fig 4A and 4B), of which rotundine displayed the most sensitive IC_50_ value (IC_50_ = 701±207nM). These ligands also inhibited 5-HT evoked cAMP generation through Sm.5HTR_L_ (Fig D in S1 Text). However, none of these compounds directly affected biosensor luminescence or cell viability of untransfected cells of at screened dosages (Fig E in S1 Text).

### Direct interrogation of Sm.5HTR

While the above data provide proof of principle for interrogation of a flatworm GPCR against a compound library in a miniaturized format, we were also curious to use the assay to investigate the properties of specific ligands prioritized by our prior work. First, we were interested in profiling specific ergot alkaloids on the basis of observations showing these compounds act as efficacious modulators of flatworm physiology [27,33,34]. Certain ergot alkaloids inhibit schistosomule contractility, while others stimulate hyperactivity [13]. In regenerating planarians, the ergopeptide bromocriptine evoked bipolarity at concentrations 100-fold less than PZQ [14], implying a potency of this class of agents against flatworm bioaminergic receptors. However, the structure-activity relationships (SAR) of ergots at flatworm GPCRs and relative selectivity over human receptors is unknown. Second, we have previously suggested an ergomimetic quality to PZQ action, raising the possibility that PZQ itself acts as a direct ligand of flatworm bioaminergic receptors likely as a serotonergic antagonist [13]. Therefore, screening for PZQ activity against Sm.5HTR was also investigated. Third, Hs.5HTR7 displays a property of pseudo-irreversible antagonism, where a subset of ligands effect a persistent inactivation of the receptor persistent beyond the duration of drug exposure [25,26]. Is this phenomenon conserved at Sm.5HTR? Finally, guided by the chemical library data, we performed a secondary screen of compounds structurally related to ‘hits’ from the initial drug screen (‘SAR by commerce’). Each of these experiments are discussed in turn below.

**Fig 5:**
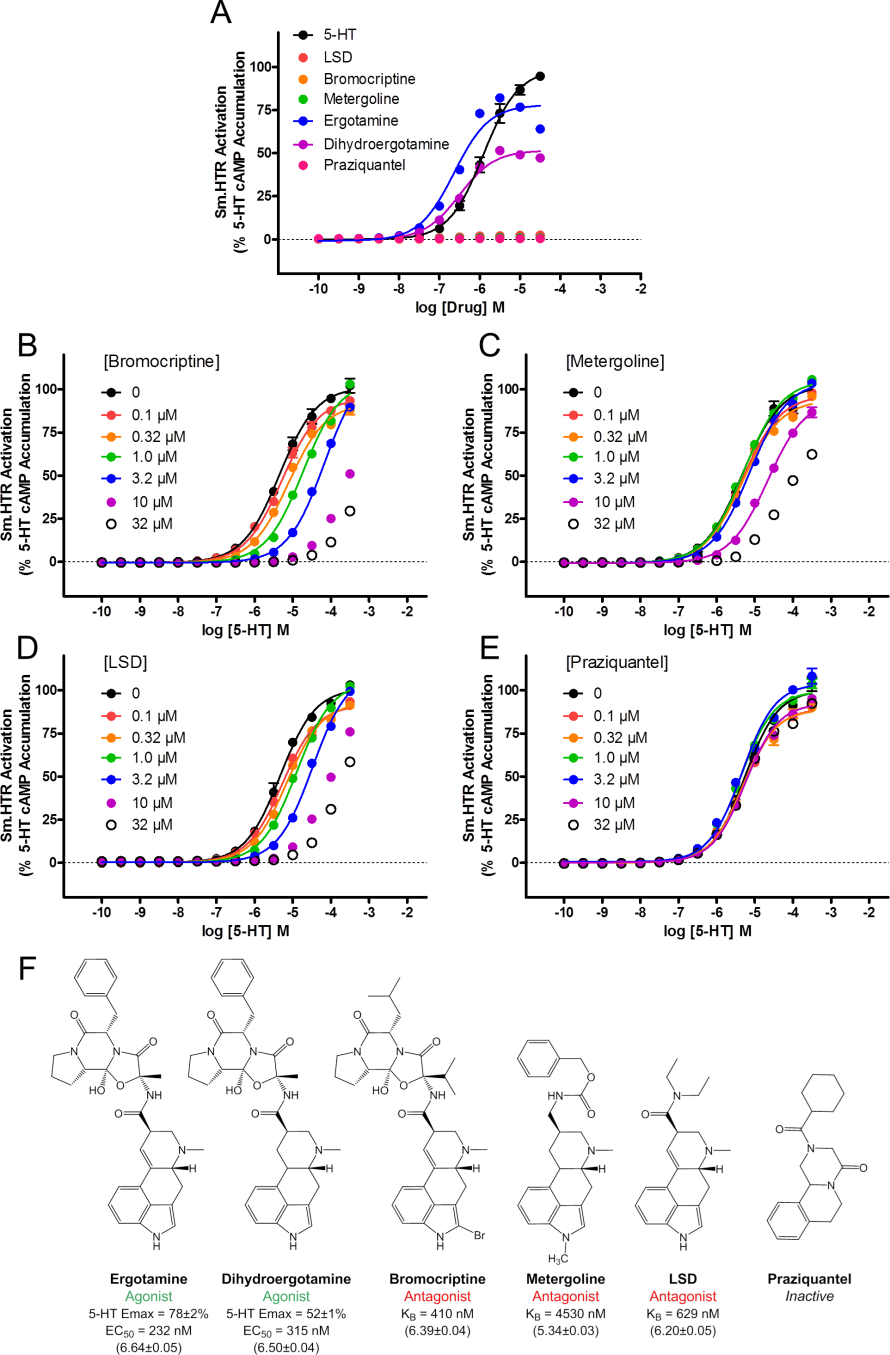
Effects of ergot alkaloids on Sm.5HTR. (**A**) Dose response relationship for various bioaminergic ligands reveals ergotamine and dihydroergotamine act as partial agonists at Sm.5HTR. (**B-D**) Bromocriptine (B), metergoline (C) and LSD (D) act as competitive antagonists of Sm.5HTR. (**E)** PZQ lacks antagonist activity at Sm.5HTR. **(F)** Structure-activity-relationship for various ergoline ligands at the Sm.5HTR. Data in parentheses represent pEC_50_ ± S.E.M or pK_B_ ± S.E.M.

First, is Sm.5HTR activity modulated by ergot alkaloids? Several ergot alkaloids were screened against Sm.5HTR and these experiments revealed agonist activity of ergotamine and dihydroergotamine, which have previously shown to stimulate the basal contractility of schistosomules [13]. Ergotamine and dihydroergotamine were more potent (EC_50_ of 232nM and 315nM, respectively) than 5-HT (EC_50_ of ~1μM, Fig 5A), but with a lower maximal response suggestive of partial agonism. By contrast, other ergoline ligands, bromocriptine, metergoline and the hallucinogen lysergic acid diethylamide (LSD), an agonist at vertebrate 5-HT_2A_ receptors, exhibited no efficacy at Sm.5HTR (Fig 5A). To investigate further the nature of these inactive ligands, the ability of increasing doses of bromocriptine (Fig 5B), metergoline (Fig 5C) and LSD (Fig 5D) to modulate 5-HT evoked cAMP accumulation at the Sm.5HTR was assessed. Each of these ergot ligands caused a right-shift in the 5-HT dose-response relationship consistent with competitive antagonism (Fig 5B–5D). At higher concentrations (>10μM) bromocriptine and LSD showed almost complete inhibition of 5-HT evoked cAMP generation. To quantify the extent of antagonism, a Schild regression analysis [35,36] was performed which yielded affinity constants (K_B_) of 410 nM for bromocriptine, 629nM for LSD and 4530 nM for metergoline (Fig 5F). These data show that ergot alkaloid derivatives can act as potent modulators of schistosome 5-HTRs.

Second, screening of PZQ against Sm.5HTR in this assay did not reveal any modulation of receptor activity over doses that would convey an antiparasitic effect (Fig 5A and 5E).

**Fig 6:**
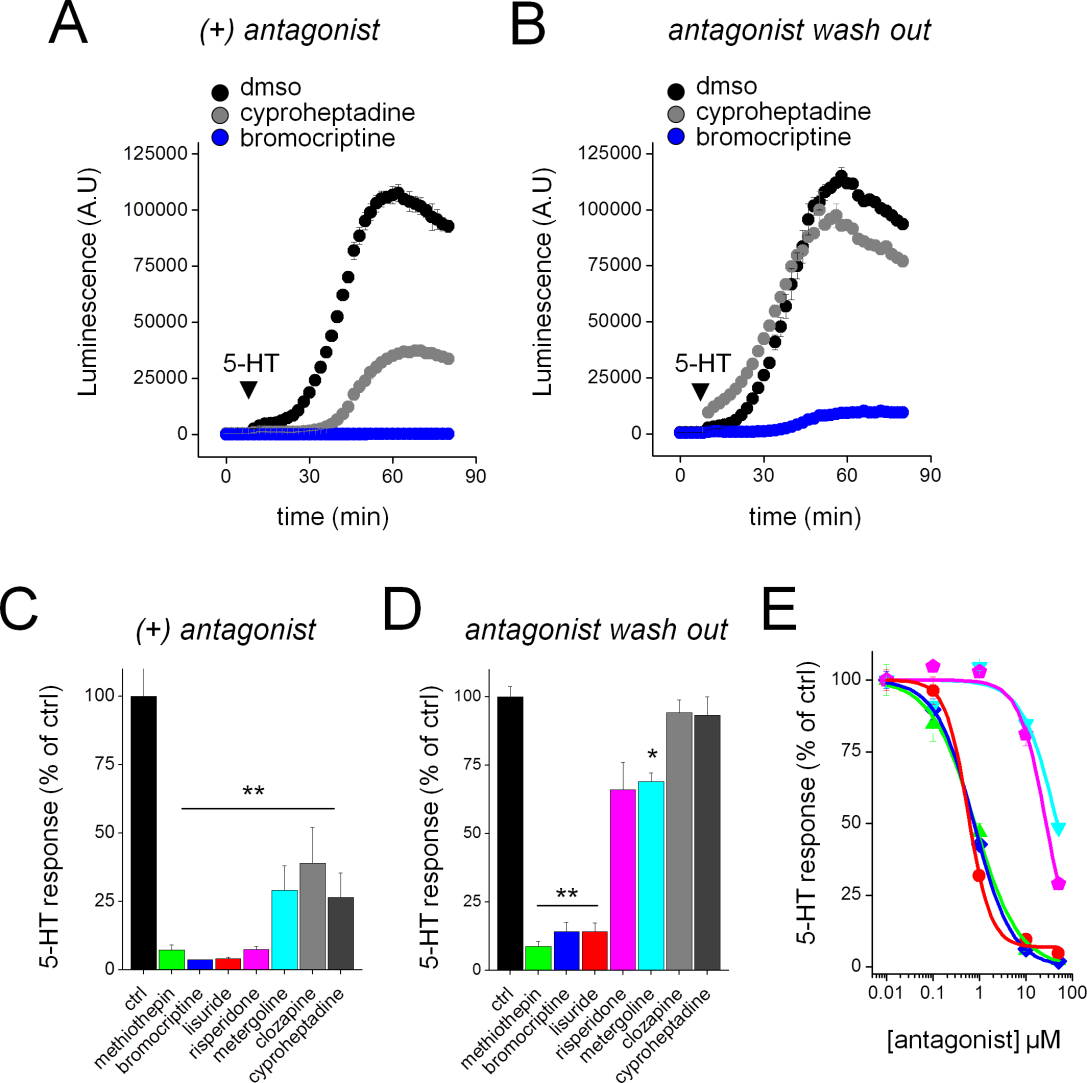
Long lasting inhibition of Sm.5HT7R evoked by a subset of ligands. Sm.5HTR displays an inactivating antagonist property reported for human 5HT7R. (**A**) Both the ‘inactivating antagonist’ bromocriptine (10μM, blue) and the competitive antagonist cyproheptadine (10μM, grey) acutely inhibit the effect of 5-HT (10μM) at Sm.5HTR (5-HT alone, black). (**B**) Sm.5HTR remains insensitive to 5-HT following washout of bromocriptine but not cyproheptadine. Cells were pre-incubated with antagonists as in (A) for 30 mins, followed by solution exchange, and the assay for 5-HT responsiveness 1hr later. (**C**) Inhibition of 5-HT response at Sm.5HTR by both ‘inactivating antagonists’ established at Hs.5HT7R (methiothepin, bromocriptine, lisuride, risperidone, metergoline) and competitive antagonists (clozapine, cyproheptadine). All drugs were tested at 10μM for 30mins. **, p <0.01. (**D**) Persistent effects of antagonists (10μM) shown in (C) after washout and subsequent assay for 5-HT response (1hr later). **, p <0.01, *, p <0.05. (**E**) Titration of these ‘inactivating antagonists’ revealed the dose-response relationship for Sm.5HTR inhibition after washout. Colors correspond to drug identity in C&D. Data represent mean±s.e.m., n = 3 (C-E).

Third, to investigate the properties of antagonists at Sm.5HTR, we compared the action of bromocriptine (a known ‘irreversible antagonist’ of Hs.5HTR7 [26]) with the competitive antagonist cyproheptadine. While both antagonists acutely inhibited Sm.5HTR function (Fig 6A), inhibition evoked by bromocriptine persisted after antagonist wash-out while cyproheptadine inhibition was fully reversed by 1 hour after ligand removal (Fig 6B). Expanding this assay to other ligands revealed long-lasting inhibition with several ligands previously established as pseudo-irreversible antagonists at Hs.5HT7R (methiothepin, bromocriptine, lisuride, risperidone and metergoline) but not with the competitive blockers clozapine and cyproheptadine (Fig 6C and 6D). The most potent ligands were bromocriptine, methiothepin and lisuride (Fig 6E). Therefore, although ligand specificities of these GPCRs are divergent, a unique aspect of receptor phenomenology is conserved between the human and parasite receptor.

Finally, we profiled compounds structurally related to those compounds prioritized from the library screen in terms of parasite selectivity. As two of these top hits were dimethoxyisoquinoline derivatives (rotundine, alfuzosin) we focused on agents containing this moiety. Slight modifications of rotundine structure were sufficient to alter the GPCR inhibition profile (Fig F in S1 Text), as reflected by comparison of berberine/palmatine (decreased potency and selectivity for Sm.5HTR) and tetrabenazine (selectivity for Sm.5HTR retained). Similarly, comparison of the closely related structures tetrandrine and berbamine suggested a discriminating structure-activity profile for Sm.5HTR (Fig F in S1 Text).

**Fig 7:**
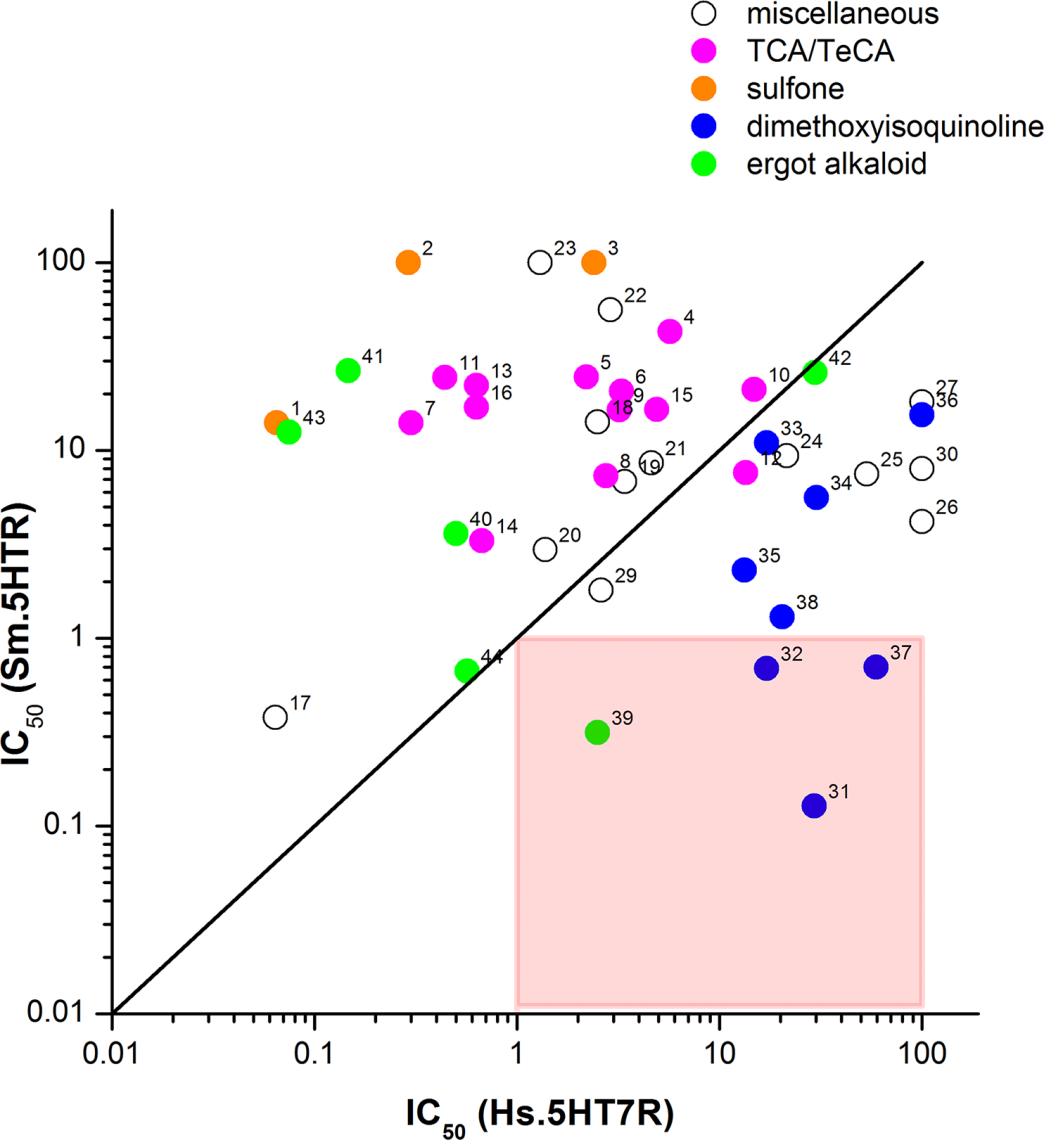
Structure activity relationships for various drug classes against Hs.5HT7R and Sm.5HTR. Comparison of IC_50_s for compounds inhibiting cAMP generation via Hs.5HT7R (abscissa) or Sm.5HTR (ordinate). Compounds with no preferential selectivity for either receptor, showing similar IC_50_s, would cluster along the solid line. Hits in the lower right quadrant (red square) show sub-μM potency at Sm.5HTR but supra-μM potency at Hs.5HT7R. Four compounds meet this criterion (bromocriptine = ‘39’, rotundine = ‘37’, tetrandrine = ‘31’ and tetrabenazine = ‘32’). Compound classes are indicated as follows: ergot alkaloids (green), isoquinolines (blue), tricyclic and tetracyclic antidepressants (magenta), sulfonyl compounds (orange), miscellaneous structures (open). Individual compounds are: 1, SB269970; 2, amisulpride; 3, SB742457; 4, olanzapine; 5, mianserin; 6, quetiapine; 7, clozapine; 8, cyproheptadine; 9, ketotifen; 10, loratadine; 11, maprotiline; 12, clomipramine; 13, desloratadine; 14, rupatadine; 15, vortioxetine; 16, amitriptyline; 17, risperidone; 18, domperidone; 19, chlorprothixene; 20, clemastine; 21, aripiprazole; 22, ketanserin; 23, ifenprodil; 24, tripelennamine; 25, fluoxetine; 26, atomoxetine; 27, orphenadrine; 28, lisuride; 29, benztropine; 30, cyclizine; 31, tetrandrine; 32, tetrabenazine; 33, berberine; 34, 6, 7-diethoxy-1, 2, 3, 4-tetrahydroisoquinoline; 35, corynoline; 36, alfuzosin; 37, rotundine; 38, fanchinoline; 39, bromocriptine; 40, metergoline; 41, LY215840; 42, nicergoline; 43, mesulergine; 44, dihydroergocristine.

Evaluation of structural data from all these assays provides insight to the structural selectivity between parasite and human receptors. Fig 7 arrays worm IC_50_ values versus human IC_50_ values, such that compounds with submicromolar IC_50_ values and selectivity for the parasite Sm.5HTR receptor fall into the bottom right quadrant. As expected, given the historical bias in ligand design for affinity toward human receptors, most compounds favor the human receptor (falling ‘above the line’ in Fig 7). For example, most of the screened tricyclic and tetracyclic antidepressants show higher affinity for Hs.5HT7R (12/13 compounds screened). Similarly, ligands with phenyl-sulfonyl groups (SB 269970, SB742457) that are potent inhibitors of Hs.5HT7R [37] (Fig 4), completely lacked activity at Sm.5HTR. In contrast, compounds exhibiting potency and selectivity toward the parasite receptor (bottom right quadrant) were the ergot alkaloid bromocriptine and several dimethoxyisoquinoline compounds revealed by our experiments (rotundine, tetrabenazine, tetrandrine).

### Effects of compounds on schistosomules

**Fig 8:**
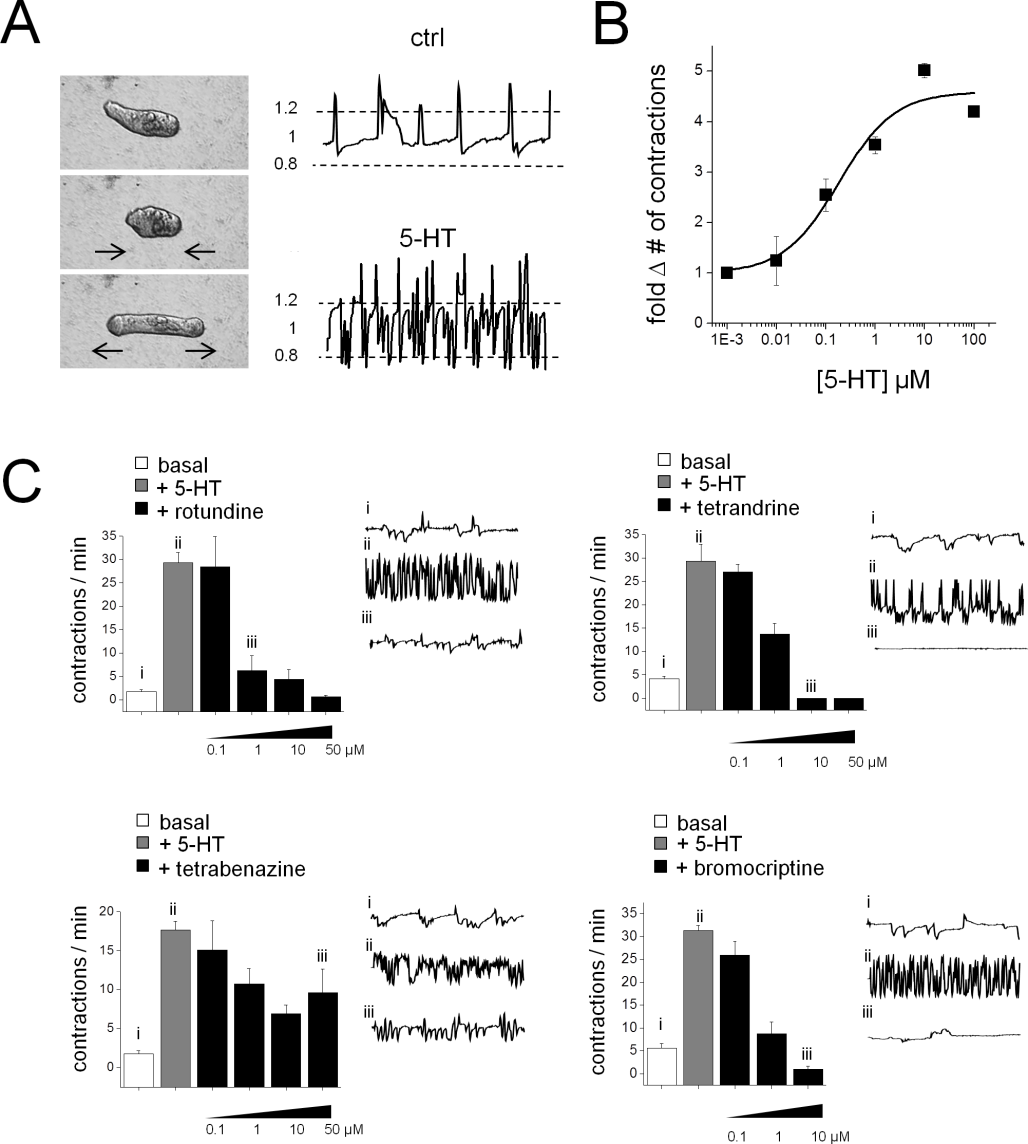
Small molecule inhibitors of Sm.5HTR antagonize 5-HT stimulation of schistosomule contractility. Effects of selected ligands on schistosomules. (**A**) 5-HT stimulates basal contractility in *S*. *mansoni* schistosomules resolved through measurements of body length over time (1 minute recording duration). A contractile cycle is defined when a deviation of ≥20% of the average body length (dashed lines) occurs. (**B**) Dose-response curve for 5-HT stimulation of contractility. (**C**) Schistosomule movement was quantified for basal mobility (i, no 5-HT addition; white bars), after addition of 5-HT ((ii, 10μM 5-HT; grey bars), and subsequent exposure to Sm.5HTR inhibitors in the presence of 5-HT (iii, indicated doses; black bars). Representative body length traces over one minute for individual schistosomules are shown for indicated conditions (right). Bar graphs represent mean±s.e.m. of independent samples, n = 3. Drugs assayed represent ligands identified as Sm.5HTR antagonists in the GPCR screen (rotundine), and follow up testing of methoxyisoquinoline compounds (tetrandrine, tetrabenazine) and the ergot alkaloid bromocriptine.

Do these hits from the Sm.5HTR screen *in vitro* translate into effectiveness against parasites? To assess this issue, selected compounds were screened for effects on schistosomule contractility. Schistosomules exhibit a basal level of spontaneous contractile activity (Fig 8A) which provides a simple phenotype for assaying neuromuscular activity. In this system, 5-HT is myoexcitatory: exogenous addition of 5-HT causes an increase in the basal contractile rate in a dose-dependent manner (Fig 8B). Subsequent addition of the four compounds validated above as potent blockers of Sm.5HTR (rotundine, tetrabenazine, tetrandrine, bromocriptine) were examined. Three of these compounds–rotundine, tetrandrine and bromocriptine–all potently inhibited 5-HT evoked contractions (IC_50_≤1μM). Tetrabenazine was however less efficacious *in vivo*, effecting only a ~50% inhibition of 5-HT evoked contractility at the highest dose (100 μM). Therefore three of the four compounds prioritized by the Sm.5HTR screening data conferred an inhibitory action against schistosomules.

**Fig 9:**
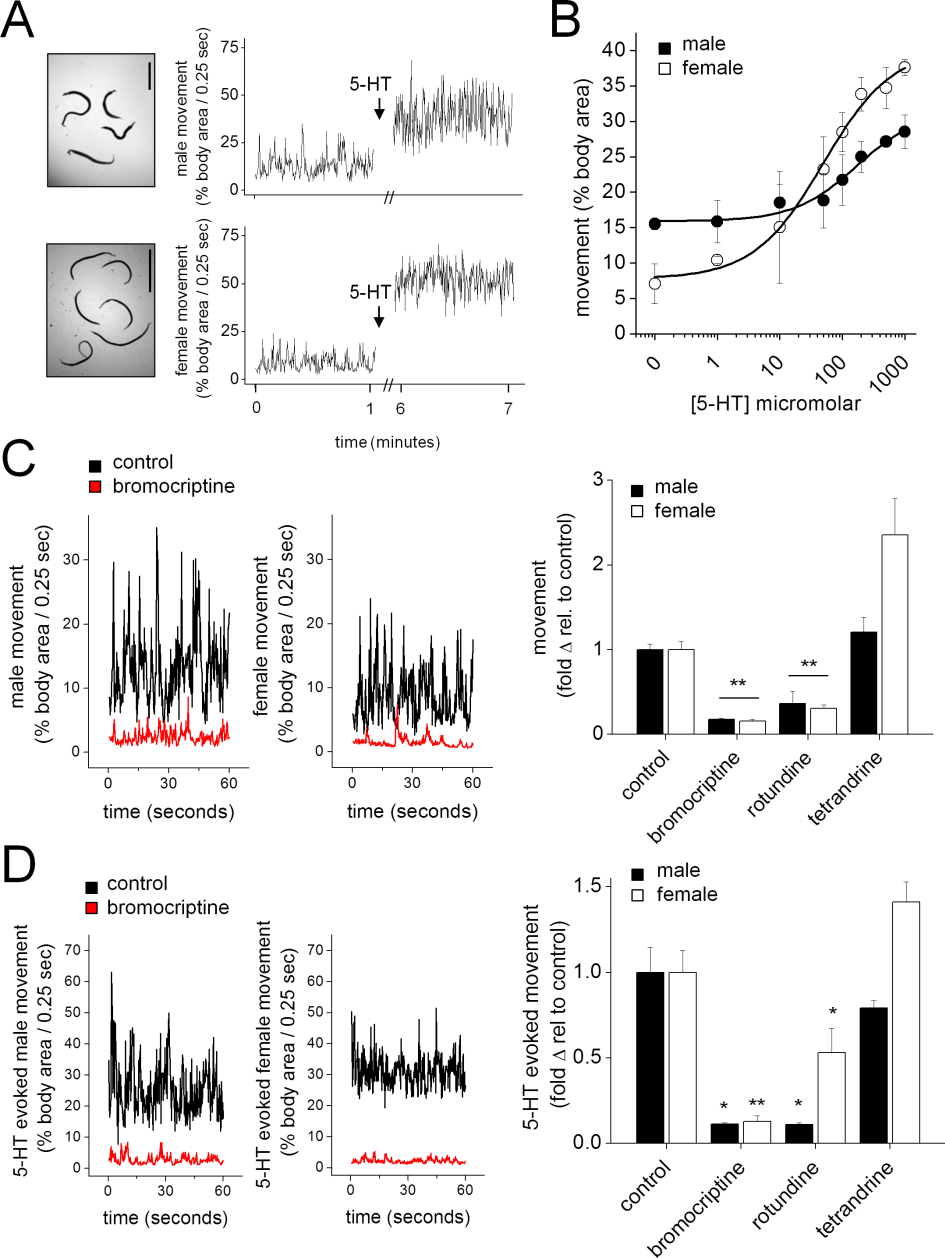
Sm.5HTR inhibitors antagonize basal and 5-HT stimulated movement in adult schistosomes. (**A**) Movement of adult male (top) and female (bottom) schistosomes under basal conditions (no 5-HT) and in the presence of 5-HT (100μM, arrow). Traces represent one minute of recorded movement for each condition. Scale, 1cm. (**B**) Dose response curves showing movement of male (solid circles) and female (open circles) schistosomes exposed to increasing concentrations of 5-HT. (**C**) Left, effect of bromocriptine (10μM) on the basal movement of adult male and female schistosomes. Representative traces showing the movement of worms in the absence of drug (black) and the presence of bromocriptine (red). Right, quantification of basal movement for male (solid bars) and female (open bars) worms exposed to the indicated compounds. (**D**) Left, effect of bromocriptine (10μM) on 5-HT (100μM) stimulated movement of adult schistosomes. Representative movement of worms exposed to 5-HT alone (black) or bromocriptine and 5-HT (red). Right, quantification of 5-HT stimulated movement for male (solid bars) and female (open bars) worms exposed to the indicated compounds. n≥3 independent experiments. * p < 0.05, ** p < 0.01.

The action of Sm.5HTR ligands were then examined against adult schistosome worms *in vitro*. Isolated worms exhibited basal mobility, and application of 5-HT significantly increased the movement of unpaired male and female worms (Fig 9A). Basal movement and the magnitude of the 5-HT evoked stimulation differed between males and females (Fig 9A). These effects was quantified by performing dose-response relationships (Fig 9B). Sex differences in the magnitude of 5-HT evoked cAMP generation [38], Sm.5HTR transcript and Sm.5HTR protein expression have previously been reported [20,39]. The action of rotundine, tetrandrine and bromocriptine were then assessed against basal (Fig 9C) and 5-HT stimulated worm movement (Fig 9D). Addition of bromocriptine and rotundine markedly inhibited worm movements at rest whereas tetrandrine enhanced movements of isolated female worms (Fig 9C). Bromocriptine and rotundine also inhibited the 5-HT evoked increases in worm movement, and again tetrandrine lacked an inhibitory effect (Fig 9D). From these experiments, we conclude bromocriptine and rotundine also act as effective paralytics of adult schistosome worms.

**Fig 10:**
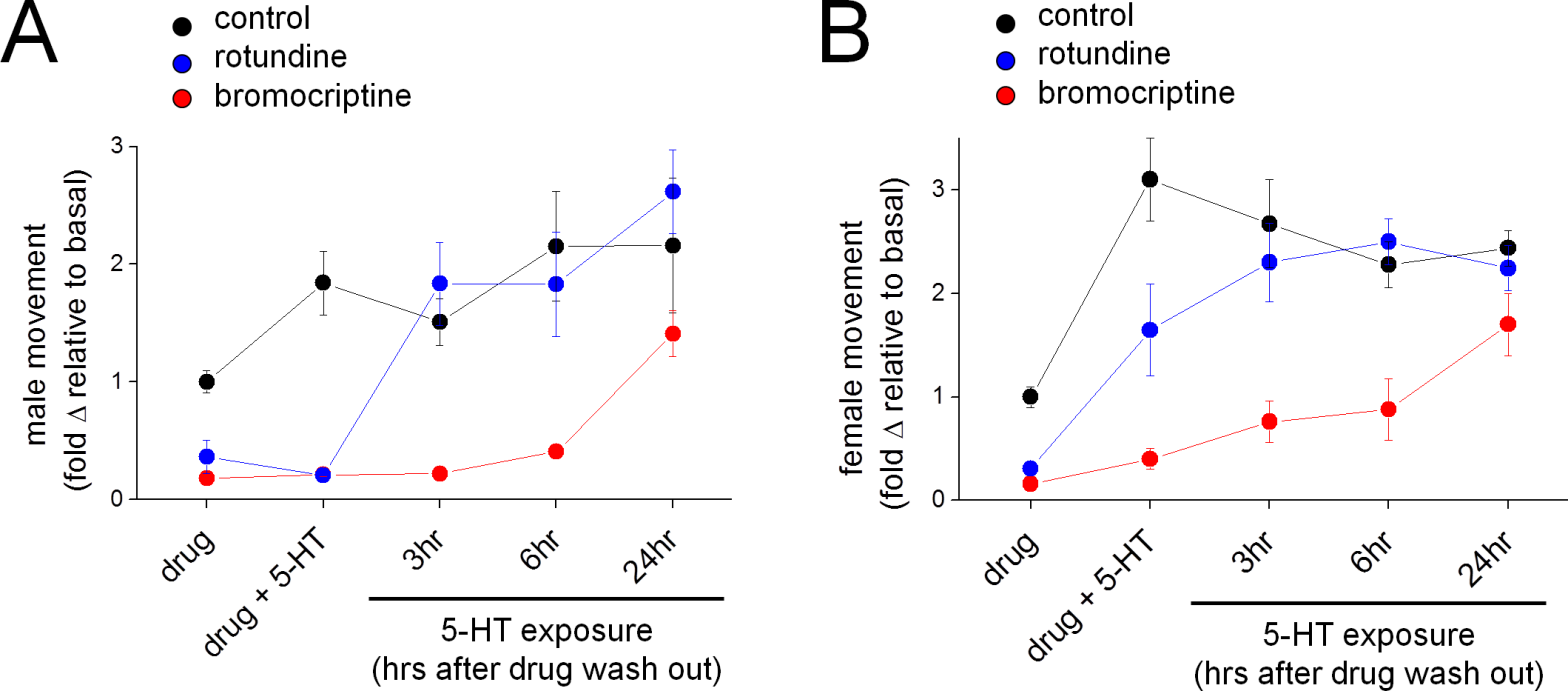
Long lasting inhibition of schistosome movement caused by bromocriptine. Time course of schistosome recovery from exposure to Sm.5HTR antagonists (black = DMSO control, blue = rotundine, red = bromocriptine). Mobility of worms was recorded following exposure to antagonist (10μM, ‘drug’, 2 hour exposure) and addition of 5-HT (100μM, ‘drug + 5-HT’). Media was then exchanged, and recordings were subsequently made on the same worms stimulated with 5-HT (100μM) at the indicated timepoints (3, 6, 24hrs after drug washout). Data for males (A) and females (B) is presented normalized to basal movement of control (dmso) worms in the absence of 5-HT.

Finally, we were interested in examining the kinetics of inhibition caused by bromocriptine and rotundine to probe whether the protracted inhibition of Sm.5HTR observed *in vitro* (Fig 6) was manifest *in vivo*. To do these experiments, worms were exposed to bromocriptine or rotundine, and then challenged with 5-HT at various points after drug removal. As expected both drugs inhibited basal worm movement, and blunted 5-HT evoked stimulation (Fig 10). The time course of reversal of these effects was then examined. For male worms, which were effectively paralyzed by both drugs (Fig 9C), the paralytic effects of rotundine were reversed within 3hrs of drug exposure (Fig 10A). However worms exposed to bromocriptine remained impaired for considerably longer, with recovery of movement being only demonstrable 24hrs after bromocriptine removal (Fig 10A). A similar timecourse of recovery from bromocriptine exposure was also resolved for female worms (Fig 10B). These data suggest that bromocriptine exposure evokes a protracted paralysis of adult schistosomes.

## Discussion

In this study we executed a proof of principle pilot screen to evidence the feasibility of pharmacological profiling a flatworm GPCR in HTS format. The assay system employed relied on an genetically encoded luminescent biosensor [23]. This is an appealing approach as this strategy is non-destructive and affords the ability to continually monitor the kinetics of cAMP generation from a single sample. Further by directly reporting cAMP levels, rather than transcriptional outcomes (e.g. cAMP reporter genes), this approach also reveals proximal receptor activity in real time to help discern how specific compounds are modulating GPCR activity. Acceptable Z’ scores were reliably obtained in 384 well format (Fig A in S1 Text), and we note the sensitivity of this approach has permitted responses from endogenous GPCRs to be resolved even in ultra-high throughput screening formats (3456-well plates). Obviously, this particular biosensor is suited only for G_s_ and G_i_-coupled GPCRs, but the optimization of other biosensors—for example, genetically-encoded Ca^2+^ indicators [40], or reporters that are agonist independent [41]—should aid HTS profiling of other flatworm GPCRs coupling to different second messenger cascades.

### Differences between human and schistosome 5-HT7 receptors

The importance of unbiased profiling of flatworm GPCR targets is underscored by visualization of the entire dataset (Fig 3E) that underscores a divergence in ligand specificities between the schistosome 5-HT receptor (Sm.5HTR) and the closest human homolog (Hs.5HT7R, ~30% amino acid identity). This divergence in ligand specificity evidences concerns over use of established mammalian ligands to infer flatworm physiological mechanisms as many chemical probes used to study human 5HT_7_ receptors have modest effects on Sm.5HTR at similar concentrations. Examples include the sulfonyl derivatives SB269970, SB742457 and amisulpiride (Fig 4). The fact that 5-HT receptors in different organisms have evolved divergent characteristics profiles is of course unsurprising: the adult schistosome lives within the human host circulatory system, a 5-HT rich environment, where it continuously ingests and cycles 5-HT replete cells. The characteristics of the ligand binding site of Sm.5HTR may therefore necessitate adaptations to this niche. While this pharmacological divergence may limit repurposing efforts for existing serotonergic ligands that have been optimized toward human usage, it is nevertheless encouraging for *de novo* ligand discovery if pharmacological differences between flatworm and human receptors can be exploited to selectivity target parasite biology.

In this regard, our data identified several ligands were identified with a preference for Sm.5HTR over Hs.5HT7R, and reciprocally several ligand classes were deprioritized owing to an observed preferential selectivity for the human receptor (‘above the line’ in Fig 7B). These latter groupings included the sulfonyl derivatives discussed above, as well as tricyclic and tetracyclic antidepressants which have previously been shown to cause schistosomule hyperactivity [42], likely through inhibition of monoamine transporters [43]. Although features of these drugs that convey potency in schistosomules have been identified [42], these features do not necessarily convey selectivity (over Hs.5HT7R, or human 5-HT transporters). A similar case could be made for many ergot alkaloids, with the noted exception of bromocriptine, which was the only ergot screened to date exhibiting higher selectivity (~10-fold) for the parasitic 5-HT receptor (Fig 7). We had previously shown bromocriptine to displace ^3^H-dopamine in planarian binding assays [14], but clearly bromocriptine also possess potent anti-serotonergic properties in flatworms consistent with the polypharmacology of ergot alkaloids. The antagonist effect of LSD against Sm.5HTR (Fig 5B) was also unexpected, given LSD action as a serotonergic agonist in mammals. Further attention is needed to identify features of the ergoline scaffold that convey preferential modulation of parasitic 5-HTRs, given the ~100-fold range in IC_50_s observed (Fig 7).

In contrast to the observed human bias of many ligands, several compounds with preferential selectivity toward Sm.5HTR were resolved (Figs 4B and 6B). First, several modulators of biogenic amine transport, represented by compounds such as atomoxetine and fluoxetine. Fluoxetine is a serotonin reuptake inhibitor while atomexetine, a non-halogenated derivative of fluoxetine, is employed as a norepinephrine transporter inhibitor. Compounds of this class are known to block 5-HT GPCRs [44]. Second, and perhaps most striking, were ligands containing dimethoxyisoquinoline moieties (blue, Fig 7), several of which exhibited clear bias toward Sm.5HTR. These included two compounds with prior precedent as bioaminergic blockers—alfuzosin, a mammalian adrenergic (μ1) blocker, and rotundine which inhibits dopamine and serotonin binding at D1, D2 and 5-HT_1A_ GPCRs [45]. Rotundine potently inhibited Sm.5HTR (IC_50_ ~700nM) while lacking any effect on Hs.5HT7 at concentrations up to 50μM. Chemical exploration of this scaffold is therefore further warranted, especially in light of the small number of compounds screened in this study. We also note that the stalwart anthelmintic PZQ is also a isoquinoline derivative, although direct interrogation of PZQ against Sm.5HTR did not yield any effect (Fig 5A and 5E). This does not preclude the possibility that PZQ acts as a ligand at another flatworm bioaminergic GPCR [13], one explanation for the functional antagonism observed between PZQ and 5-HT in planarians [13], schistosomules [13] and adult schistosomes [28,46].

Subsequent screening of bromocriptine (the most parasite selective ergot alkaloid, Fig 7), and the three promising isoquinoline ‘hits’ against both schistosomules and adult worms (Figs 8 and 9) revealed a clear inhibition of 5-HT evoked hypermotility from two of the four compounds (bromocriptine, rotundine) prioritized from the screen of heterologously expressed Sm.5HTR. The two other Sm.5HTR ligands (tetrabenazine, tetrandrine) were poorly effective. Such attrition of leads is expected when advancing candidates identified *in vitro*. For example, the stimulatory action of tetrandrine against adult worms (Fig 9), not observed in the schistosomule dataset (Fig 8), may reflect a counteracting stimulatory action at other schistosome GPCRs upregulated at the adult stage. Differences in GPCR expression [39] may also contribute to the observed differences in drug and 5-HT action between male and female worms (Fig 9). Overall, this was an encouraging translation from *in vitro* data to activity against different parasite life cycle stages, supporting the rational design and development of antiparasitic drugs aimed at schistosomal GPCRs.

### Similarities between human and schistosome 5-HT7 receptors

Despite the divergence in pharmacological selectivity between the human and schistosome 5-HT GPCRs, it is worthwhile highlighting an important similarity between these receptors which may prove a boon for anthelmintic development. The human Hs.5HT7R is induced into a prolonged inactivated state by exposure to a subset of ligands, termed ‘inactivating antagonists’ [25,26]. These inactivating antagonists are structurally diverse and include the ergot alkaloid bromocriptine, risperidone, methiothepin, lisuride and metergoline [26]. Application of these ligands caused a prolonged inactivation of Hs.5HT7R activity in heterologous expression systems or in assays on endogenous receptors [25,26,47]. Data suggests this aspect of receptor phenomenology may be conserved with Sm.5HTR, the most abundant deorphanized GPCR in adult schistosome worms, when evaluated in receptor level (Fig 6) and whole organism assays (Fig 10). The predominant expression of this specific GPCR in this organism, together with conservation of this receptor property, provides a clearly targetable weakness for anthelmintic development. If transient exposure to an inactivating antagonist inhibits parasite mobility well beyond the pharmacokinetic persistence of the drug within an infected individual, this would be clearly be effective for antiparasitic action and serve to minimize dosing requirements in challenging healthcare environments. Sm.5HTR is also expressed at multiple life cycle stages, and is conserved in other PZQ-sensitive parasites. Further exploration of this property and identification of parasitic-selective ligands that convey this effect are warranted, and such activities will be facilitated by the approaches optimized in this study.

In conclusion, these data demonstrate the optimization and application of a real-time biosensor assay for interrogating flatworm GPCRs *in vitro*, which is capable of scaling to HTS. Application of this approach to profile Sm.5HTR revealed parasitic-selective ligands and ligand series, as well as conservation of a ligand-evoked inactivation mechanism at the most predominantly expressed *S*. *mansoni* 5HTR.

## Materials and Methods

### Compounds & reagents

Serotonin (5-HT), 3-Isobutyl-1-methylxanthine (IBMX) and DMSO were purchased from Sigma Aldrich. The GPCR Compound Library was purchased from Selleck Chemicals (Catalog No. L2200) pre-dissolved in DMSO (10mM). 5HT_7_ ligands DR4485, LY215840, metergoline and 5-Carboxamidotryptamine (5-CT) were purchased from Tocris Bioscience. Methoxy-isoquninoline alkaloids (rotundine, tetrabenazine, berbine, palmatine, tetrandrine and berbamine) were purchased from Sigma Aldrich, while fangchinoline was purchased from AK Scientific.

### Cell culture and 5-HT receptor expression

HEK293 cells (ATCC CRL-1573.3) were cultured in DMEM supplemented with 10% fetal bovine serum (FBS), penicillin (100 units/ml), streptomycin (100 μg/ml), and L-glutamine (290 μg/ml). Cells were transiently transfected (ViaFect, Promega) as per the manufacturer’s protocol at a density of 2x10^6^ cells per T-25 cell-culture flask. Cells were transfected with plasmids encoding GloSensor (Promega) and either Sm.5HTR (Smp_126730, GenBank accession number KF444051.1) or Sm.5HTR_L_ (KX150867), bothGPCRs being codon optimized for mammalian expression, or Hs.5HT7a (GenBank accession number NM_000872.4, R&D Systems) subcloned into pCS2(-). Cell culture reagents were from Invitrogen. Epitope tagged constructs were used to verify expression, and untagged constructs used for all luminescence assays.

### Western blotting

HEK293 cells were transfected with Sm.5HTR subcloned into a pCS2(-) mammalian expression vector possessing an NH_2_-terminal 6xmyc tag and harvested 24 hours post-transfection. Cell pellets were solubilized in 1% NP-40, protein was quantified using Bradford reagent (Pierce). Denatured sample (10 μg) was run on a Mini-PROTEAN TGX Precast Gel (BioRad) at 150V. Semi-dry transfer to PVDF membrane was performed using a Trans-Blot Turbo Mini-PVDF Transfer pack (Bio Rad) at 25V for 30 minutes. The membrane was blocked with 5% nonfat milk in TBST (Tris-buffered saline, 0.1% Tween 20) for 1hr at room temperature, incubated with anti-myc antibody overnight at 4°C (Santa Cruz, 1:500 dilution in 5% milk -TBST) prior to washing in TBST (3x10 minutes) and incubation with secondary antibody (LiCor Goat anti-mouse IRDye 800, 1:5000 dilution in 5% milk -TBST) for 1hr at room temperature. After washing (3x10 minutes in TBST), membranes were visualized on a LiCor Odyssey imaging system.

### Molecular cloning

Sequence for Sm.5HTR_L_ was determined by cloning from an *Schistosoma mansoni* cDNA library (adult male and female NMRI strain, BEI cat #NR-36056) using high fidelity Advantage HD DNA polymerase (Clontech) and primers described in [20]. PCR products were ligated into the pGEM-T Easy vector system (Promega) prior to DNA sequencing. Additional sequence contained in the Sm.5HTR_L_ isoform was verified by 5’/3’ RACE (SMARTer RACE Kit, Clontech) using total RNA extracted from *S*. *mansoni* (Trizol reagent, Ambion). Products were cloned into the pRACE vector (In-Fusion HD Cloning Kit, Clontech) prior to DNA sequencing.

### GloSensor cAMP assays

For assays performed on adherent cells, HEK-293 cells were transferred one day post transfection to 96 well, solid white plates (Corning, cat # 3917) coated with 0.01% poly-L-lysine (Sigma Aldrich) at a density of 5 x 10^4^ cells / well in DMEM supplemented with 1% dialyzed FBS (Gibco). After overnight culture (37°C/5% CO_2_), media was decanted and replaced with 100μL / well HBSS supplemented with 0.1% BSA, 20mM HEPES (pH 7.4) and GloSensor reagent. Plates were allowed to equilibrate at room temperature for two hours prior to performing luminescence assays (GloMax-Multi Detection System plate reader, Promega). Conditions for individual assays were described as per figure legends. The standard assay to detect changes in cAMP utilized the F22 sensor in media supplemented with IBMX (200μM). Ligands were added at a concentration of 20x per well for experiments (i.e. 5μl of drug solution added to 100μl of cells). A dose of 5-HT corresponding to the [EC_80_] for the relevant receptor used for all antagonist screens. The average standard deviation of the 5-HT E_max_ in internal, vehicle treated control wells (at least 8 per plate) was 13% for Sm.5HTR and 8% for Hs5HT7R. For the resazurin reduction assay for cellular viability, cells were incubated with resazurin (final concentration, 10μM) and tested ligands with fluorescence measurements (560 nm excitation/590 nm emission) made at 1.5hr intervals.

To test putative irreversible antagonists, assays on suspension cells were performed one day post transfection. Cells in a T-75 flask were trypsinized (0.25% w/v) and transferred to a 14mL tube, centrifuged at 300 RCF, and resuspended in HBSS supplemented with 0.1% BSA, 20mM HEPES. Compounds were added at 10 μM, and after 30 minute incubation at room temperature cells were centrifuged (300 RCF, 5 minutes) and resuspended in fresh media. This wash step was repeated, and cells were resuspended in HBSS supplemented with 0.1% BSA, 20mM HEPES and GloSensor reagent. Cells were gently rotated to prevent aggregation and settling over the course of the two hour equilibration period, after which time they were transferred to 96 well plates at a density of 8 x 10^4^ cells / 100μL per well and assays described for adherent cells.

### Schistosomule assays

*Biomphalaria glabrata* (M-line) snails exposed to *Schistosoma mansoni* miracidia (Strain PR-1) were obtained from BEI Resources (Cat. number NR-21961) and cercaria were shed following exposure to light (1.5 hours). Cercaria were manually transformed into schistosomula by vortexing (3 x 45 seconds, each separated by 3 minutes on ice) and tails were removed by gradient centrifugation (24ml Percoll, 4ml 10X EMEM, 1.5ml penicillin-streptomycin, 1ml of 1M HEPES in 0.85% NaCl, 9.5ml distilled water) at 500g/15min at 4°C. Supernatant containing tails was discarded, and schistosomules were resuspended in Basch media and incubated (37°C, 5% CO_2_) overnight before conducting mobility assays. For contractility experiments, somules were incubated in 5-HT free Basch media to resolve a basal contractility rate. To establish a dose response curve for 5-HT, serial dilutions of 5-HT were added to Basch media and somule contractile frequency recorded. In order to assess the effects of antagonists on somule movement, recordings were made of cohorts in 5-HT free Basch media, media supplemented with 5-HT (10μM), and media supplemented with both 5-HT (10μM) and the drugs indicated in Fig 8. Schistosomules were incubated in 24 well plates (~200 schistosomules / 0.5mL media per well) for 30 minutes (37°C/5% CO_2_) prior to acquiring videos of schistosome movement (1 minute recordings / well) using a Nikon Coolpix 5700 camera affixed to a Nikon Eclipse TS100 microscope (10x objective). The WrmTrck plugin for ImageJ was used to quantify worm mobility [21]. Briefly, the major axis of each schistosomule body length was extracted from the raw output of WrmTrck and an average length was determined for the duration of the recording. Contractions were quantified by determining the number of episodes during which the worm body length deviated from the average by over 20%. *S*. *mansoni* protocols were approved by the Iowa State University Institutional Biosafety Committee.

### Adult schistosome mobility assays

Female Swiss Webster mice exposed to *Schistosoma mansoni* cerceria (Strain PR-1) at 5–7 weeks old were obtained from BEI Resources (Cat. number NR-34792) and sacrificed 6–8 weeks post-infection. Mature *S*. *mansoni* were harvested from the mesenteric vasculature by portal perfusion [48]. Briefly, mice were anesthetized in a CO_2_ chamber and sacrificed by cervical dislocation. Mice were perfused with sodium citrate (25mM) and adult schistosomes were harvested from the mesenteric veins. Schistosomes were washed in RPMI media containing penicillin (1000 units/mL), streptomycin (1000 μg/mL) and 25mM HEPES, then transferred to RPMI media supplemented with 2mM glutamine and 5% heat inactivated FBS. Worms were incubated overnight at 37°C, 5% CO_2_ before conducting assays. Recordings of adult schistosome movement were captured using a Zeiss Discovery v20 stereomicroscope and a QiCAM 12-bit cooled color CCD camera. Videos were acquired at a rate of four frames per second for one minute. Recordings of female worms were acquired at 7.6x magnification, 30 mm field of view and recordings of male worms were acquired at a 5.1x magnification, 45 mm field of view. Analysis was performed in ImageJ as described previously [20]. Briefly, image (.tiff) stacks of each recording were imported and processed by converting to binary format so that pixel measurements represent area of the worms’ bodies. Mobility was quantified by measuring the difference in pixels resulting from subtracting the value of one frame (n) from those of the next frame in the sequence (n+1). This difference was expressed as a percentage of the pixels in the initial frame (n), providing a measurement of the worms’ movement over a period of 0.25 seconds. This calculation was performed for each frame in the video, and the results were averaged over the length of the recording. Values reported represent the mean (±) standard error of at least three independent experiments. Animal work was carried out with the oversight and approval of the Laboratory Animal Resources facility at the Iowa State University College of Veterinary Medicine.

## Supporting Information

S1 Text **Figure A**. Sm.5HTR GloSensor luciferase assay in 384 well plate format. (**A**) HEK293 cells transiently transfected with Sm.5HTR and the F22 cAMP biosensor were assayed for changes in luminescence in response to addition of 5-HT (10μM, arrow). (**B**) Dose response curve depicting luminescence values assayed 60 mins following 5-HT addition. (**C**) Z’-scores over time illustrated for the representative experiment shown in (A). All experiments shown performed in the presence of IBMX (200μM). **Figure B.** Sm.5HTR response to bioaminergic ligands. (**A**) Response of HEK293 cells transfected with the F22 GloSensor to various Class A GPCR ligands (10μM), revealing a lack of responsiveness to serotonergic ligands but robust cAMP generation in response to catecholamines acting on endogenous HEK293 cell GPCRs. (**B**) Response of HEK293 cells co-expressing Sm.5HTR and the F22 biosensor to various serotonergic and monoaminergic ligands previously identified to lack activity on endogenous G_s_ coupled GPCRs. **Figure C.** Comparison of ligand class specificities against Sm.5HTR and Hs.5HT7R. Categorized ligand specificities of individual compounds that block Sm.5HTR and Hs.5HTR7 from classification index of the screened library. While Sm.5HTR and Hs.5HTR7 show distinct selectivity profiles to the 23 and 31 ligands identified as ‘hits’, the broader classification of these ligands is similar. **Figure D.** Effect of Sm.5HTR antagonists on Sm.5HTR_L_. Luminescence response from Sm.5HTR_L_ expressing HEK293 cells to 5-HT (EC_80_ dose = 0.8μM) in the presence of indicated antagonists (10μM). Data are shown relative to control samples unexposed to antagonist (black). Antagonist compounds screen encompass compounds from the GPCR library screen (grey), methoxy-isoquinolines (open), and ergot alkaloids (striped).**Figure E.** Toxicity test for screened compounds. (**A**) HEK293 cells transiently transfected with the F22 cAMP biosensor were incubated with test compounds (10μM, 30 mins) and then assayed for forskolin (20μM, 30 mins) evoked cAMP generation. Tested ligands showed no effects on luminescence signal values. The mitochondrial complex I inhibitor rotenone served as a positive control. (**B**) Resazurin reduction assay for cell viability of HEK293 cells exposed to test compounds (10μM) and resazurin (10μM) for 3 hours at 37°C. Fluorescence was measured using a 560nm excitation/590nm emission filter set. Sodium azide was used as a positive control. **Figure F.** Effects of selected compounds on Sm.5HTR and Hs.5HT7R evoked cAMP generation. (**A**) Inhibition curves shown for the following methoxy-isoquinoline related compounds against Sm.5HTR (blue) and Hs.5HT7R (green): (i) rotundine (data reproduced from Fig 4A), (ii) palmatine, (iii) berberine and (iv) tetrebenazine. (**B**) Comparison of 5HTR selectivity between (i) the tetrandrine and (ii) berbamine, a structurally related compound.
(DOCX)
