# Supplementary material for: A Miniaturized Screen of a Schistosoma mansoni Serotonergic G Protein-Coupled Receptor Identifies Novel Classes of Parasite-Selective Inhibitors
Source: PLoS Pathog. 2016 May 17;12(5):e1005651. doi: 10.1371/journal.ppat.1005651 (PMC4871480; doi:10.1371/journal.ppat.1005651)
Supplement: S1 Text — Figure A. Sm.5HTR GloSensor luciferase assay in 384 well plate format. (A) HEK293 cells transiently transfected with Sm.5HTR and the F22 cAMP biosensor were assayed for changes in luminescence in response to addition of 5-HT (10μM, arrow). (B) Dose response curve depicting luminescence values assayed 60 mins following 5-HT addition. (C) Z’-scores over time illustrated for the representative experiment shown in (A). All experiments shown performed in the presence of IBMX (200μM). Figure B. Sm.5HTR response to bioaminergic ligands. (A) Response of HEK293 cells transfected with the F22 GloSensor to various Class A GPCR ligands (10μM), revealing a lack of responsiveness to serotonergic ligands but robust cAMP generation in response to catecholamines acting on endogenous HEK293 cell GPCRs. (B) Response of HEK293 cells co-expressing Sm.5HTR and the F22 biosensor to various serotonergic and monoaminergic ligands previously identified to lack activity on endogenous Gs coupled GPCRs. Figure C. Comparison of ligand class specificities against Sm.5HTR and Hs.5HT7R. Categorized ligand specificities of individual compounds that block Sm.5HTR and Hs.5HTR7 from classification index of the screened library. While Sm.5HTR and Hs.5HTR7 show distinct selectivity profiles to the 23 and 31 ligands identified as ‘hits’, the broader classification of these ligands is similar. Figure D. Effect of Sm.5HTR antagonists on Sm.5HTRL. Luminescence response from Sm.5HTRL expressing HEK293 cells to 5-HT (EC80 dose = 0.8μM) in the presence of indicated antagonists (10μM). Data are shown relative to control samples unexposed to antagonist (black). Antagonist compounds screen encompass compounds from the GPCR library screen (grey), methoxy-isoquinolines (open), and ergot alkaloids (striped).Figure E. Toxicity test for screened compounds. (A) HEK293 cells transiently transfected with the F22 cAMP biosensor were incubated with test compounds (10μM, 30 mins) and then assayed for forskolin (20μM, 30 mins [file ppat.1005651.s001.docx]

**S1 Text**

**Figure A**.

**Figure B**

**Figure C**

**Figure D**

**Figure E**

**Figure F**
